# A musculoskeletal model of low grade connective tissue inflammation in patients with thyroid associated ophthalmopathy (TAO): the WOMED concept of lateral tension and its general implications in disease

**DOI:** 10.1186/1471-2474-8-17

**Published:** 2007-02-23

**Authors:** Roy Moncayo, Helga Moncayo

**Affiliations:** 1 WOMED, Karl-Kapferer-Strasse 5, A-6020 Innsbruck, Austria

## Abstract

**Background:**

Low level connective tissue inflammation has been proposed to play a role in thyroid associated ophthalmopathy (TAO). The aim of this study was to investigate this postulate by a musculoskeletal approach together with biochemical parameters.

**Methods:**

13 patients with TAO and 16 controls were examined. Erythrocyte levels of Zn, Cu, Ca^2+^, Mg, and Fe were determined. The musculoskeletal evaluation included observational data on body posture with emphasis on the orbit-head region. The angular foot position in the frontal plane was quantified following gait observation. The axial orientation of the legs and feet was evaluated in an unloaded supine position. Functional propioceptive tests based on stretch stimuli were done by using foot inversion and foot rotation.

**Results:**

Alterations in the control group included neck tilt in 3 cases, asymmetrical foot angle during gait in 2, and a reaction to foot inversion in 5 cases. TAO patients presented facial asymmetry with displaced eye fissure inclination (mean 9.1°) as well as tilted head-on-neck position (mean 5.7°). A further asymmetry feature was external rotation of the legs and feet (mean 27°). Both foot inversion as well as foot rotation induced a condition of neuromuscular deficit. This condition could be regulated by gentle acupressure either on the lateral abdomen or the lateral ankle at the acupuncture points gall bladder 26 or bladder 62, respectively. In 5 patients, foot rotation produced a phenomenon of moving toes in the contra lateral foot. In addition foot rotation was accompanied by an audible tendon snapping. Lower erythrocyte Zn levels and altered correlations between Ca^2^^+^, Mg, and Fe were found in TAO.

**Conclusion:**

This whole body observational study has revealed axial deviations and body asymmetry as well as the phenomenon of moving toes in TAO. The most common finding was an arch-like displacement of the body, i.e. eccentric position, with foot inversion and head tilt to the contra lateral side and tendon snapping. We propose that eccentric muscle action over time can be the basis for a low grade inflammatory condition. The general implications of this model and its relations to Zn and Se will be discussed.

## Background

The pathogenesis of orbital changes seen in patients with thyroid associated orbitopathy (TAO) has been matter of research since decades [1,2]. One theory that has eluded confirmation is the proposal of a low level inflammation of the connective tissue [3-5]. The original idea put forth in 1995 by the research group of Jack R. Wall [3] has found limited resonance in the scientific literature (citation analysis done with SCOPUS, Elsevier B.V.). On the other hand, the topic of low grade inflammation is found more frequently in relation to the metabolic syndrome, to abdominal obesity and to cardiovascular disease as well as in relation to decreased muscle mass and elevated cytokine levels, e.g. of IL-6 and TNF [6-9]. A common denominator found both in TAO as well as in these metabolic situations is the elevation of cytokines [10-13]. It is interesting to emphasize, that effective treatment of the thyroid condition related to TAO, i.e. hyperthyroidism, does not lead to a normalization of IL-6 levels in these patients [11] which suggests that IL-6 might not originate from the thyroid [14]. On the other hand, physiology research has demonstrated that muscle tissue can indeed produce IL-6 and TNF [15]. In spite of this fact, the role of peripheral skeletal muscle function has been seldom evaluated in TAO. None the less muscular changes have been quantified in thyroid diseases showing a situation of diminished strength [16-18]. Therapeutical consequences, however, have not been derived from these dynamometric findings.

Elevation of glycosaminoglycans (GAGs) has also been described to occur in TAO [19,20], however the mechanisms linking GAGs to inflammation are intricate [21] and elevated GAGs can be found in patients with other autoimmune diseases [22]. In cases of recurrent of eye disease levels of GAGs can again be elevated [23]. A recent publication on TAO concluded that "the elevations in urinary GAGs in these patients appear to reflect widespread stimulation of fibroblast metabolic processes, as might occur in the setting of systemic connective tissue inflammation" [24]. Mechanisms leading to GAGs elevation in relation to connective tissue can be the scarring of fasciae [25] as well as endoplasmatic reticulum (ER) stress [26]. GAGs are also related to the extra cellular superoxide dismutase system (EC-SOD) [27]. SOD has been found to be elevated in GD, and remains so even after an euthyroid state has been achieved [28].

Due to the involvement of extra ocular muscles (EOM) in TAO, investigators have also studied the role of these tissues as a potential antigenic target. Based on serological methods, putative muscle antigens have been identified in EOM as well as in peripheral muscle tissue [29,30]. On clinical grounds, Graves' disease can present features such as myalgia, swelling of the calves and fasciitis [31]. Symptomatology of amyotrophic lateral sclerosis has also been reported in association with thyroid disease [32-34]. The evaluation of muscle strength in hyperthyroid patients has revealed a situation of reduced strength which can be regained after therapy [16,18]. In 1977 Delporte et al. found an association with musculoskeletal components such as with polymyositis [35,36]. In another study an eosinophilic fasciitis was found [37]. In 1977 one report described manifestations of muscular and neurological origin in hyperthyroidism. The authors called the situation hyperthyroid myopathy, a condition which was not necessarily proportional to the hyperthyroid state, and proposed also a polysystemic nature of Grave's disease [38]. Finally, motor neuron alterations have also been described in connection with hyperthyroidism [34,39].

Two recent findings clearly emphasize the relation of TAO to musculoskeletal structures. In the first place antibodies directed against a muscle antigen which is involved in Ca^2+ ^release, i.e. myocardial calsequestrin, have also been described [40]. Ca^2+ ^release is an immediate regulator of muscle contraction [41-43]. In the second place, antibodies directed against collagen type XIII have also been found [44]. Collagen type XIII is found at the myotendinous junctions [45].

Besides these clinical and serological aspects, imaging methods have also provided hints regarding the involvement of musculoskeletal structures. Recently it has been shown that muscles of patients with Graves' disease present increased peripheral glucose utilization, i.e. ^18^F-fluorodeoxyglucose uptake [46]. Increased peripheral glucose metabolism can either signalize inflammatory changes [47] or increased tissue metabolism such as in oncological conditions [48]. Indirect evidence on the presence of inflammation in muscles of TAO patients, e.g. neck muscles and shank, can be seen through the use of radioactive labeled octreotide, a somatostatin analog [49,50]. Furthermore it should be kept in mind that posture – a neuronal and musculoskeletal function – is intimately related to gaze [51,52], and gaze is altered in TAO. Again, these lines of evidence suggest an interaction with the musculoskeletal system in TAO. One possible explanation for the scarcity of clinical evaluations in this direction is the lack of sufficient clinical expertise in the field of musculoskeletal diseases [53,54].

Considering the compelling amount of evidence mentioned above it appears that both the musculoskeletal system and the connective tissue system can be involved in TAO. The aims of the study were to analyze and characterize musculoskeletal elements in TAO patients using a simple functional manual clinical examination method. The elements in this analysis were derived from features of biomechanical functions that relate the feet to eye function [51,52,55]. The presence of potential somatic dysfunction was studied using a propioceptive stretch test [56]. Finally erythrocyte levels of Zn and Cu, which are closely related to the function of superoxide dismutase (SOD) [57-59], as well as the levels of Mg and Ca^2+^, which are important in muscle action, were also analyzed.

## Patients and Methods

The clinical and laboratory study included 13 patients with TAO, 12 females and 1 male, age range 10–46 years and 16 controls, 11 females and 5 males, age range 15 to 58 years. According to the Werner classification [60], all patients were in stages I or II. Mild signs of inflammatory activity in the form of conjunctivitis were present in 3 cases. All patients provided written consent for participation and for their identities to be revealed. At the time of the investigation, latent hyperthyroidism was present in 5 patients. Informed consent was provided. The investigation was carried out in accordance with the Declaration of Helsinki. The institutional ethics committee approved the study. The investigation was done by endocrinologists trained also in musculoskeletal methods (Applied Kinesiology) as well as in acupuncture. Additional expertise on sports physiology and physical training was contributed by RM (Master of Advanced Studies Health and Fitness, University of Salzburg, Austria). Neither specialized biomechanical methods nor a biomechanical research facility were available.

**Figure 1:**
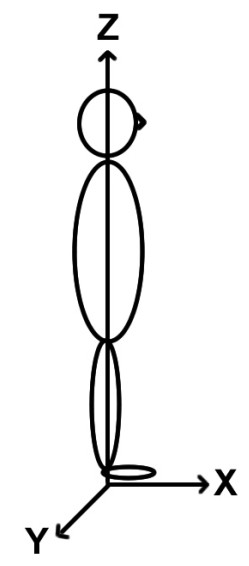
Description of the axis definitions used in the study. The positive x direction was the forward orientation, the positive y direction was the rightward orientation, and the positive z direction was the upward orientation

The initial clinical examination started with the patient sitting and then standing in front of the investigator. For the observations on posture the 3D coordinates were defined as follows: the positive x direction was the forward orientation, the positive y direction was the rightward orientation, and the positive z direction was the upward orientation (Figure 1). Some facial characteristics such as the inter-pupillary angle and the head on neck orientation [61] were quantitatively evaluated using digital photographic material.

Symmetry of foot movement during gait was observed in the frontal plane. For this the patient was asked to walk an aisle of 10 meters length. After observing the gait pattern the patient was asked to repeat the procedure and to stop on command during the stance phase of the representative side. While standing the angular deviation of the foot in relation to the straight direction of motion was measured. This procedure was adapted from the method described by Schmid [62].

The last part of the examination was conducted with the patient in an unloaded supine position where the orientation of the legs and of the feet was evaluated. This unloaded supine position has been described as being adequate for the mobilization of the stretch reflex pathway [63] where propioceptive stimuli from both the skin and the joint are activated [64]. The clinical testing was done using passive inversion and active rotation of each foot. The effect of the stretch procedures was evaluated using standard methods of Applied Kinesiology (AK) as described elsewhere [65,66]. In this context, the propioceptive provocation can elicit a so-called therapy localization (TL) which provokes a change in muscle tonicity as documented by EMG analysis [67]. The change in muscle tonicity can be evidenced by the manual examination [68]. The response pattern can thus be reduced to binary elements, 0 = absent, or 1 = present. The identification of a TL site indicates a local structural change. Based on the experience gained through the investigation and treatment of the index case of this model two acupuncture points were chosen for the correction of the TL by applying acupressure on them. The points correspond to elements of the bladder and gall bladder meridians, Bl62 or Shen mai, and GB26 or Dai mai, respectively, which have been recently shown to be related to tendinomuscular structures by in-vivo MRI imaging of gold acupuncture needles inserted at these sites (submitted to BMC in March 2006). The examination procedure is summarized in Table 1.

Laboratory examinations included the determination of erythrocyte levels of Zn, Cu, Mg, Ca^2+^, Na, K, and Fe. The determinations were carried out at a specialized laboratory (Labor Bayer, Stuttgart, Germany). In a previous study we had demonstrated that patients with thyroid disease present lower levels of Se as compared to non-thyroid disease controls (submitted to BMC, 2006). For this reason this parameter was not evaluated again in this study.

## Results

### Description of the posture characteristics

**Figure 2:**
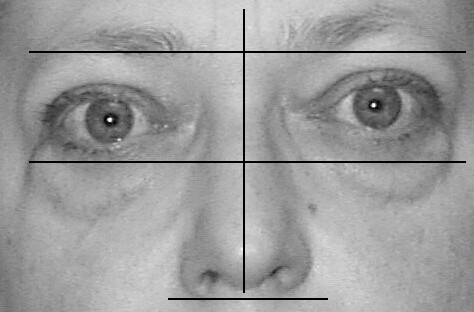
A TAO patient showing moderate changes in facial symmetry. Misalignments of the eyes on the orbit and of the orbit on the head are noted.

**Figure 3:**
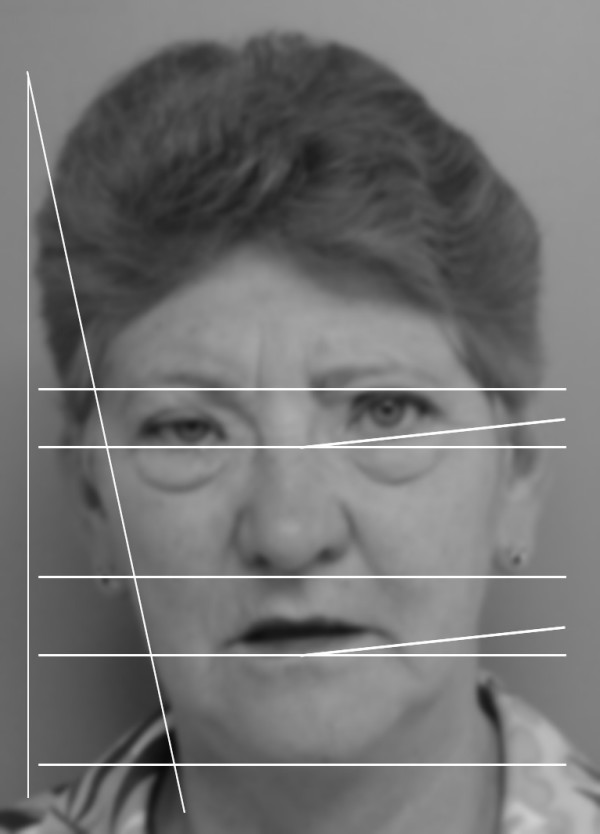
A TAO patient showing complex changes of facial symmetry. These include Y+ changes in the head on neck position, of the orbital axes as well as on the alignment of the eyes on the orbit.

**Figure 4:**
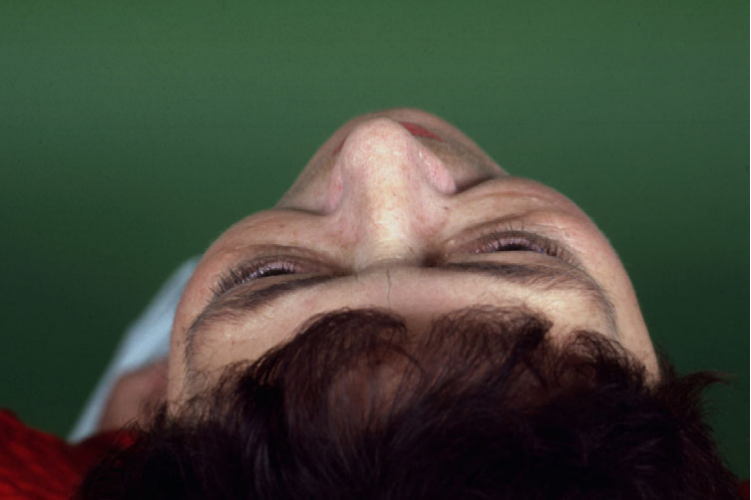
TAO patient showing left rotation the orbits and head as seen by photographic documentation taken from above.

**Figure 5:**
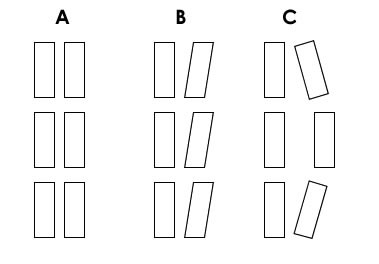
Description of the foot orientation patterns observed during gait. The left panel shows a symmetrical gait. The middle panel shows a lateral foot deviation. The right panel signalizes a rotating, semicircular pattern of foot advancement.

**Figure 6:**
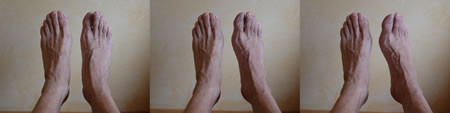
Description of the changes in foot alignment while lying supine in an unloaded position. The left panel shows a symmetrical position. The middle panel shows a slight displacement of the Y axis. The right panel shows a more pronounced displacement of the foot in the Y axis corresponding to inversion.

The visual inspection of orbital morphometry revealed several features of facial asymmetry. These changes included displacements of the inclination of the eye fissures and altered position of the eyes in the orbit as can be seen in Figs. 2, 3, 4. The evaluation of photographic material of the head revealed a head inclination in relation to the absolute vertical line ranging from 3° to 12°, mean 5.7°. The inter pupillary inclination in relation to the absolute horizontal line was 2° to 18°, mean 9.1°. During the observation of the gait pattern the controls showed a symmetrical foot orientation pattern whereas TAO patients did not. TAO patients presented 2 types of alteration: 1) an asymmetrical step pattern with one foot oriented outwards, i.e. Y+, raging from 18° to 40°, mean 27°, and 2) a swinging semi-circular step pattern (not quantified) which is shown schematically in Figure 5. In the unsupported supine position the foot angle orientation was asymmetrical in all TAO patients having one dominant side, i.e. the same one having the Y+ deviation during gait. The foot angle deviation ranged from 38° to 65°, mean 50.9°. Complex deviations of the foot orientation involving the Y+ and the Z- direction were not quantified. These changes are shown schematically in Figure 6.

### Description of the functional testing

**Figure 7:**
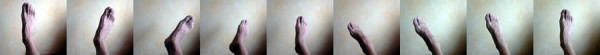
Description of the foot rotation test by which several muscle groups are activated. Besides the muscle activation propioceptive stimuli arise from skin and tendon stretching.

Using the propioceptive stretch stimulus on the feet, i.e. passive inversion, a TL reaction was seen in 4 of 16 controls and in 11 of 13 TAO patients. Active rotation of the feet (Figure 7) was positive in 3 of 16 controls and in 12 of 13 TAO patients. The finding of a positive TL was independent from the measured degree of axial displacement of the feet. Active rotation of the ankle was accompanied by a snapping sound of the joint in all TAO patients. In addition 5 out of 13 TAO patients presented an involuntary movement of the toes of the contra lateral foot being tested during foot rotation. The TL effect of these stimuli could be eliminated by gentle acupressure of 2 seconds duration on the pre-selected acupuncture points. The TL arising from passive foot inversion was neutralized in 90% of cases by the Dai mai point or Gb26 (lateral abdomen), while the foot rotation TL was neutralized mostly by the Shen mai point or Bl62 (lateral ankle), even in the cases presenting the moving toes. The correction pattern was applicable to both controls and patients.

### Analysis of the biochemical parameters

The biochemical parameters showed a diminution of the erythrocyte levels of Zn (p = 0.05) and an increased Cu to Zn ratio (p < 0.05) (Table 2). The controls showed a negative correlation between Ca^2+ ^and Mg and a positive correlation between Mg and Fe. In the TAO patients these correlations had changed. TAO patients showed a direct correlation between Ca^2+ ^and Cu while Fe was negatively correlated with Ca^2+^and Cu (Table 3). The negative correlation between Mg and Ca^2+ ^was not found in TAO patients.

## Discussion

**Figure 8:**
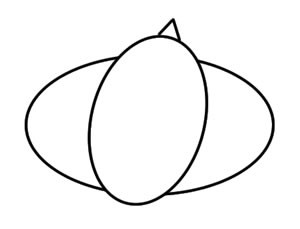
Schematic representation of the head on neck on trunk postural changes showing head rotation in a Y+ direction. Besides this rotation, lateral head tilting can also be present.

**Figure 9:**
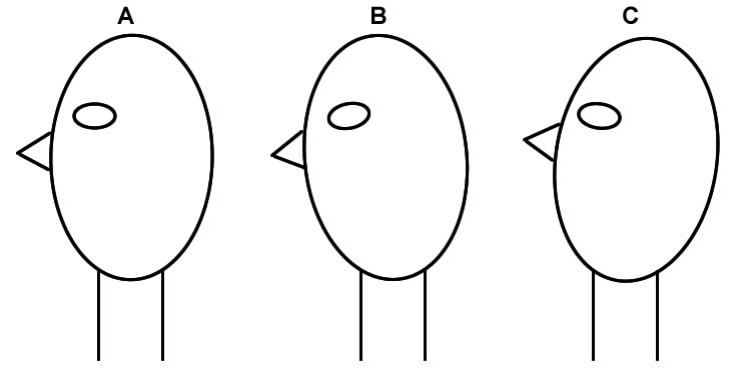
Schematic representation of the pitch changes of the head on neck position.

Based on the close relation between vision and posture control [51,52] we hypothesized that elements related to biomechanical features of posture and gait could be present in TAO patients and that such changes could be related to a low grade systemic inflammation [3,38]. In this study we have been able to show that TAO patients present facial asymmetry with deviation of the inter-pupillary axis as well as of the head-on-neck position (Figures 8, 9) together with axial rotation of the legs and feet. Active foot rotation was found to be coupled to an altered neurological response producing the phenomenon of moving toes in the contra-lateral foot. Until now, moving toes have only been described in neurological patients [69-73]. Due to the lack of a resting phase in the contra lateral foot, one can expect that propioceptive stimuli produce altered signals in the brain, thus affecting the areas involved in their coordination [74,75]. This feature is new in TAO.

**Figure 10:**
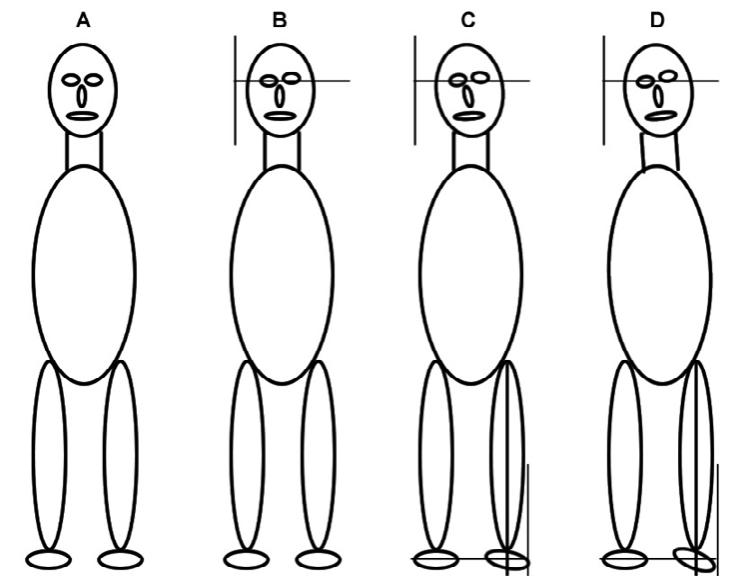
Schematic summary of the WOMED concept of lateral tension. The four panels describe the graded development of lateral tension. Panel A shows the symmetrical body position. Panel B includes only Y+ tilting of the head. Panel C includes Y+ tilting, alteration of the orbits and slight eccentric position of the leg and foot. Panel D shows the complex pattern with head, orbit, face and lower limb asymmetry. We propose that in this stage eccentric muscle action will induce low grade inflammation.

**Figure 11:**
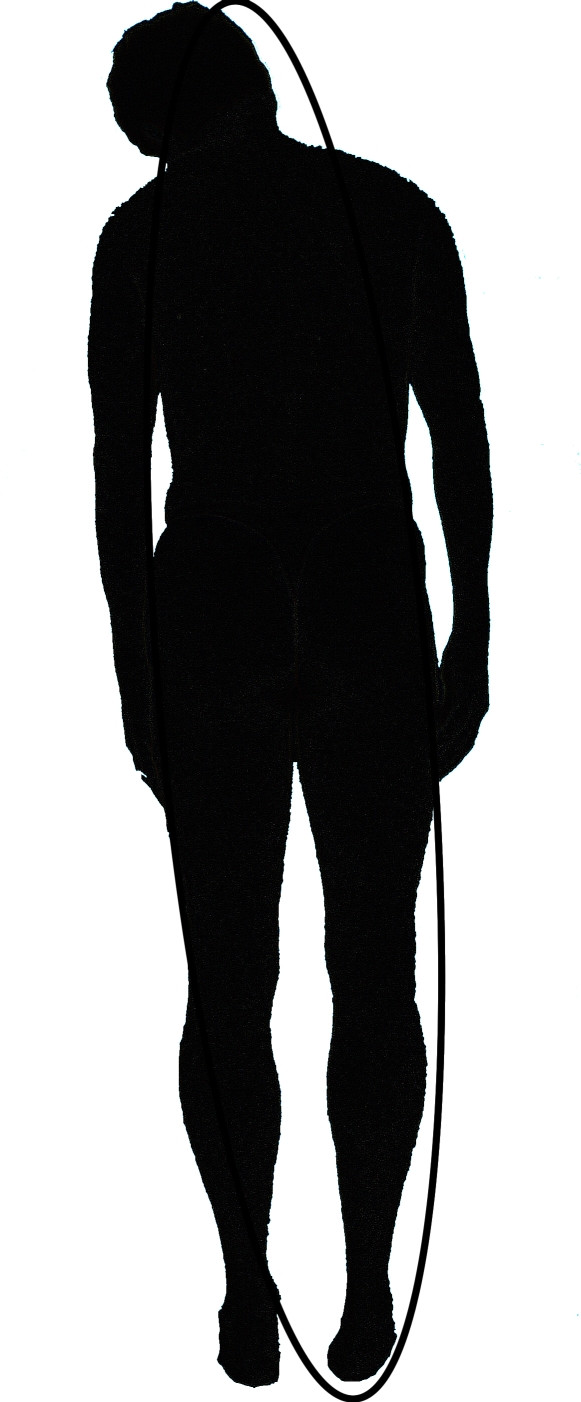
Simulated patient photography demonstrating the lateral tension concept. The basic elements of the model are included.

In addition to the moving toes phenomenon, active foot rotation or foot inversion were associated with an altered propioceptive response during manual examination which could be compensated by using acupressure on 2 specific acupuncture points. The manual examination procedure constitutes a simple binary evaluation approach where either altered musculoskeletal regulation is found or not. Based on our results we propose that TAO patients present alterations of whole body axial changes as shown in Figure 10. The most complex situation of axial misalignment resembles an arch-type body posture which we have called lateral tension. This term represents the eccentric muscle positions of the foot and leg, as well as that of the neck muscles due to a contra-lateral head tilt (Figure 11). Recent publications by the group of Milias et al. [76,77] include a photograph of normal subject that presents the typical outward rotation of the foot as we describe here. These studies were centered on the actions of Se on muscle function tests and included dynamometric analysis. This postural alteration, however, was not characterized by the authors. Literature evidence on the potential interactions of these musculoskeletal changes on posture is summarized in Table 4.

On the biochemical side, our study has confirmed the finding of diminished erythrocyte Zn levels in hyperthyroidism [78,79]. Zinc plays an important role in the maintenance of multiple body functions. One general function is that of providing site-specific antioxidative protection together with Se [80]. In a recent study we have also demonstrated that patients with thyroid disease have diminished levels of Se (submitted to BMC, 2006). We will mention some aspects that appear relevant to TAO. Low erythrocyte levels of Zn are characteristic of hyperthyroidism and not of subacute thyroiditis [81], thus suggesting that different pathogenetic mechanisms are involved in these entities. Zn availability is a key factor for the development and function of the immune system [82-84]. Fraker et al. have pointed out the importance of Zn supplementation in order to overcome "the dismantling of the immune system" [85]. Studies on TAO have seldom considered Zn even though it is an important regulator of retinal function [86-88]. Neuritis of the optic nerve has also been described as being associated with low Zn levels [89]. Smoking, which is a known risk factor for TAO [90,91], can lead to diminished Zn levels in serum [92]. Muscle and tendon function are also related to Zn. Zn uptake has been located to the connective tissue space of muscle [93]. Lack of Zn could diminish collagenase activity leading to a hardening of tendon tissue resulting in the characteristic snapping we have documented. Regarding the other biochemical parameters, we have not found any studies that have analyzed erythrocyte Ca^2+ ^and Mg levels in TAO patients. Further implications of these findings will be discussed below.

The scientific literature has abundant data on biomechanical evaluations of gait and posture, but none has been carried out in TAO patients. In the majority of studies, normal subjects restricted through the use of a bracing apparatus have been evaluated. Under these conditions, the innate posture cannot be characterized. Quantitative approaches, i.e. dynamometry, have revealed that muscle strength is diminished in patients with hyperthyroidism. In spite of having quantitative data on muscle strength, these studies [16-18] have not attempted to derive corrective procedures as we have done. A beneficial role of muscle strength for the prevention of falls was mentioned by Brennan [16]. We will comment on this in the outlook section.

The description of new features of TAO patients arising from this study is a consequence of the philosophy of our Institution WOMED. Here we are committed to deliver an integral approach to disease in each individual. Within this frame we have previously described therapeutical measures for treating periorbital edema in TAO [67] or daily stress burden in hyperthyroidism [94]. For this reason, we have chosen to call our results and observations the WOMED concept of lateral tension. This concept includes components of musculoskeletal function, endocrine function, and nutrition.

The following discussion will expand to deal with the following topics: 1) muscle involvement in Graves' disease and TAO, 2) effects of eccentric muscle action on muscle inflammation, 3) calsequestrin and the Ca^2+ ^regulation system, 4) involvement of the connective tissue and superoxide dismutase (SOD), 5) propioception and biomechanical implications, 6) functional imaging data in relation to the model, and 7) summary of the WOMED concept of lateral tension in the context of inflammation and IL-6.

### 1) Muscle involvement in Graves' disease and TAO

**Figure 12:**
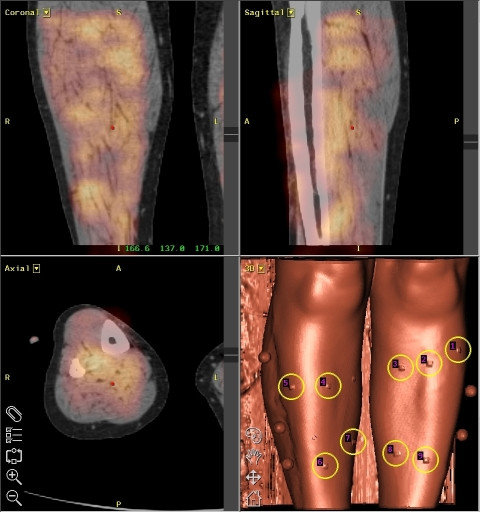
Image fusion of CT and scintigraphic data showing diffuse Octreotide uptake in the lower limbs of a patient with TAO. Octreotide uptake represents inflammatory activity. Taken from [49]. Each reconstruction plane is labeled in the upper left corner of the image. The right lower image is an anatomical reconstruction of the surface of the leg.

On clinical grounds, thyroid disease can be found to be associated with musculoskeletal components such as with polymyositis [35,36], eosinophilic fasciitis [37], as well as with myalgia and swelling of the calves and fasciitis [31]. Recent investigations have found an increased peripheral glucose utilization, i.e. ^18^F-fluorodeoxyglucose uptake in the muscles of patients with Graves' disease [46]. Increased peripheral glucose metabolism can signalize inflammatory changes [47]. Finally, indirect evidence on the presence of inflammation in muscles of TAO patients, e.g. neck muscles [50] and shank [49] (Figure 12), can be seen through the use of radioactive labeled octreotide, a somatostatin analog which can depict immunological or inflammatory sites in TAO [95]. Alterations of neck muscles can be viewed as being directly related to postural changes [96-99]. While previous investigations in TAO have focused on the EOM, it should be recalled that there are large differences in muscle mass between the EOM and the limb muscles. EOM have a volume of 3–5 ml [100,101], while the muscle mass of the legs together with abdominal muscle can amount to 15–20 kg [102,103]. It follows, that biochemical markers of inflammation such as GAGs [19,20] are more likely to come from peripheral muscles. The postural changes we describe here correspond to an eccentric muscle action of peripheral muscles. The consequences of eccentric action will be discussed in the following section.

### 2) Effects of eccentric muscle action on muscle inflammation

**Figure 13:**
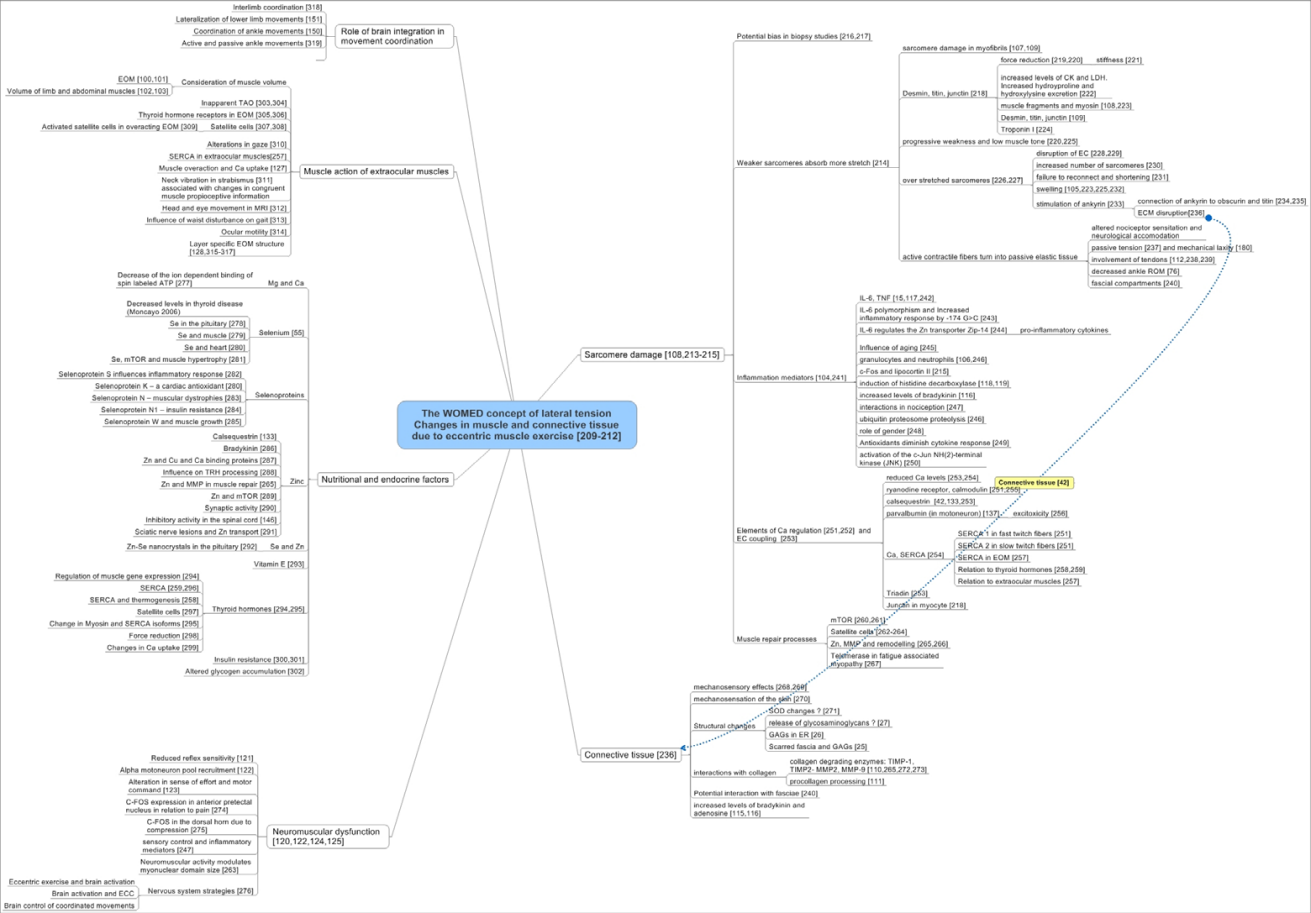
Graphical summary of relevant scientific data related to the effects of eccentric muscle action. The images recollect physiological data as well as nutritional and endocrine aspects. Emphasis is put on the roles of Zn and Se.

Investigations from the field of sports medicine have provided an ample repertoire of evidence relating eccentric muscle action to muscle inflammation, cell disruption and involvement of the connective tissue [104]. Some selected aspects include the visualization of anatomical changes such as edema by means of MRI [105], the presence of WBC in muscle [106], the disruption of the cytoskeleton and myofibrils [107-109], the involvement of collagen and of collagen degrading enzymes [110,111], the involvement of tendons [112] and fasciae [113], the release of IL-6 [114], as well as the release of bradykinin and of histamine through the induction of histidine decarboxylase [115-119]. In addition to this, neuromuscular changes and dysfunction can appear [120-125]. The phenomenon of moving toes might be related to this neuromuscular dysfunction. Finally an asymmetrical body posture will influence the mechanics of the hips [126]. Figure 13 shows an extended description of the interactions of eccentric muscle action on tissue injury and repair.

### 3) Calsequestrin and the Ca2+ regulation system

**Figure 14:**
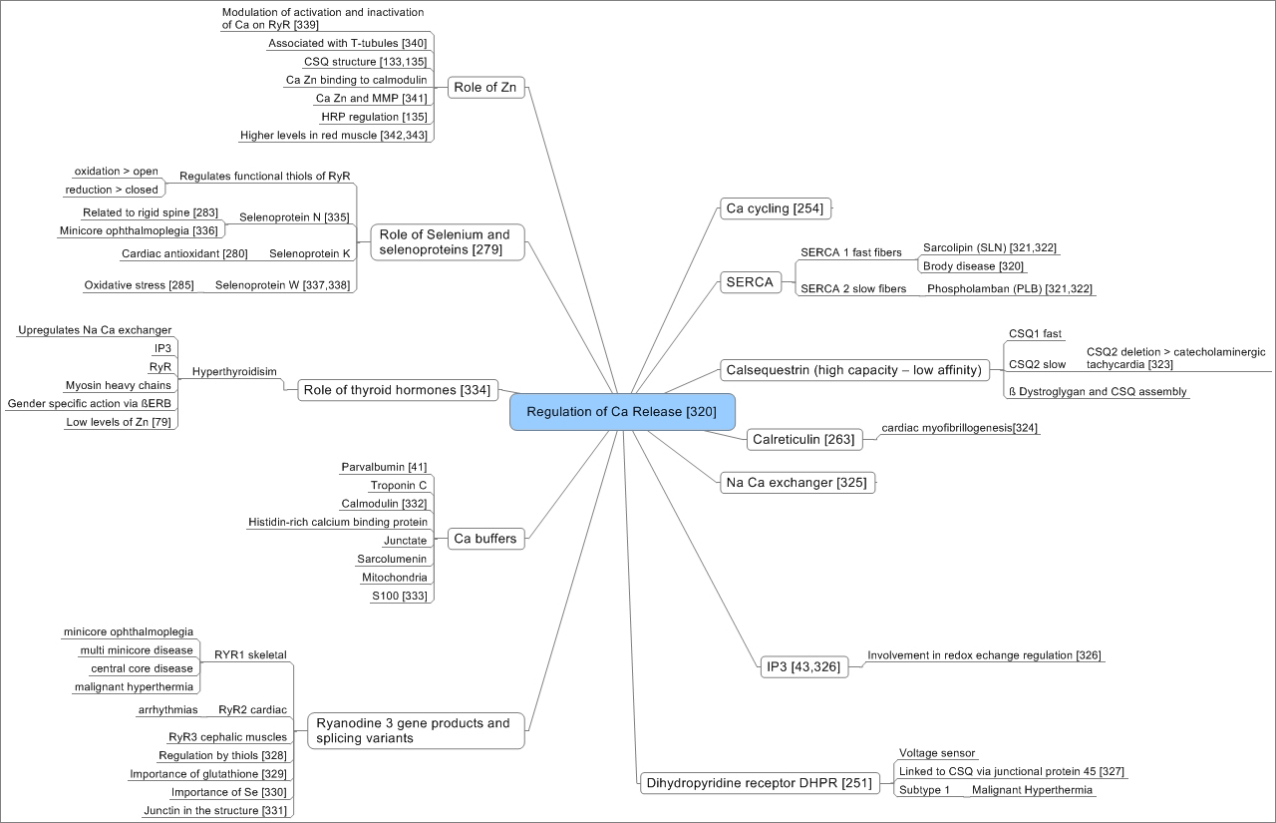
Graphical summary of relevant scientific data related to Ca regulation. Ca regulation has been suggested to be important in TAO. This assumption is based on the finding of antibodies directed against calsequestrin [40,370]. Interactions with Zn and Se are included.

In order to understand the potential impact of antibodies directed against cardiac calsequestrin (CSQ) as shown by Gopinath [40] were carried out a revision of basic and current literature dealing with Ca^2+ ^regulation. The result of this literature analysis is summarized in Figure 14. CSQ represents only a part of the system of Ca^2+ ^regulation. In addition to this it must be remembered that the slow type CSQ is a cardiac element and not highly specific for EOM [40]. It follows that these antibodies might be more relevant to cardiac manifestations of thyroid disease. On the other hand functional alterations of EOM do show an involvement of CSQ [127]. Modern analytical methods have documented the heterogeneity of anatomical and functional elements of EOM which makes them unique. One example involves the structural differences of the different layers of EOM [128]. One important functional feature of EOM is that they are to be considered as tonically active muscles such as the diaphragm where function integrity depends upon the sufficient availability of antioxidant substances such as Se and SOD [129]. Another important regulator of EOM function integrity can be Ca^2+ ^[130].

We have recently shown the importance of Se in relation to thyroid antibody levels in incipient hypothyroidism: antibodies were present when Se levels were inadequately low [131]. Once Se substitution has been implemented, thyroid antibody levels will diminish. These results are similar to those presented by Turker et al. [132]. Recently it has also been demonstrated that thyroid disease patients have lower Se levels as compared to controls (Moncayo and Moncayo, submitted to BMC, 2006). It follows that antioxidative protection can be altered in TAO. Additional interactions between Ca^2+^, Se, and selenoproteins can occur through the ryanodine receptor as shown in Figure 14.

Interactions of Ca^2+ ^and Zn are known to exist in relation to calsequestrin [133]. In relation to eccentric changes due to chronic cardiac volume overload, changes in Ca^2+ ^and calsequestrin can result in altered contraction responsiveness to Ca^2+ ^[134]. A further relation to muscle function can be found in the histidine-rich Ca^2+^-binding protein which can also bind Zn and is localized in the sarcoplasmic reticulum [135]. Thus it appears that the changes in the intracellular concentration of ions and metals might be affecting muscular function. Some of these relations have been described in ALS [57,58,136,137]

### 4) Involvement of the connective tissue and SOD

As described above an eccentric muscle action represents a situation of tissue damage involving muscles, tendons, fasciae and the connective tissue. Protection against these processes can be expected to come from superoxide dismutases (SOD) [138-140]. The extra-cellular SOD (EC-SOD) has the capacity to bind several components of the connective tissue such as heparin, collagen and GAGs [141], some of which can be found to be altered in TAO [27]. The maintenance of functional SOD subtypes requires an adequate supply of Zn and Cu [142,143]. In our patients we have not been able to measure these parameters in muscle tissue, however erythrocyte levels can be taken as a surrogate compartment [59]. In scoliosis it is known that Zn levels are lower in the affected muscles [144]. Thus, the same might happen in TAO. We can only speculate that the functions of SOD might be influenced by the shift of ions and metals we have found as is known in amyotrophic lateral sclerosis (ALS). Zn deficiency can alter the SOD so that it can be neurotoxic locally [145]. Extrapolating from experimental data neurotoxicity could affect elements involved in inhibitory regulation of neural function such as the spinal cord [146,147] or cerebellar cortex [148]. In turn, cerebellar structures are involved in muscle action and coordinated muscle action [149-151]. Our finding of moving toes in TAO patients could correspond to a situation of lack of spinal cord inhibition.

### 5) Propioception of the lower extremity and general biomechanical implications

Considering that mechanical test procedures for ankle stability are liable to problems [152] we chose to use a simple manual test for the evaluation of ankle mobility based on principles of Applied Kinesiology. Dedicated research centers utilize electromagnetic devices for a similar purpose [153]. Guralnik et al., using a small battery of functional tests, have shown that lower extremity function is an important parameter for the prediction of disability in later years [154]. Within this field of prediction of disability-related problems, clinical investigations should attempt to detect alterations and implement correction measures. Our simple manual method has the advantage that once a TL has been found, the effectiveness of a correction step can be evaluated. In our study the use of acupressure on the lateral side of the abdomen or of the ankle was enough for reversing this propioceptive stimulus. Based on the observations of Roll and collaborators referring to the interactions between the dynamometric map function of the feet and posture regulation [155,156] we can corroborate this notion. Tension transmission along the foot, ankle and lower limb to the trunk belong to our WOMED concept of lateral tension (Figure 11). Tension transmission is based on the principle of fascia and fascial continuity [157]. More importantly in 1989 Roll, Vedel and Roll described the intricate relation between the retroocular muscles and posture control [55]. Lack of ankle function in relation to posture might be an important factor in the tendency to fall which turns to be a matter of serious concern in older patients. Analytical studies have revealed the importance of gait variability in relation to stride width [158]. Others have emphasized that all ankle motions contribute to the maintenance of gait balance [159]. Our rotational propioceptive stimulus includes all these conditions of ankle motions. In addition, neurophysiological investigations on the acupuncture points of the lateral side of the foot have shown a relation to the visual cortex [160,161] thus showing complementarity to the data from Roll. Altogether, the foot and ankle appear to be important determinants of gaze, posture and gait.

The area of the trunk also plays an important role in controlling the direction of gait. As has been shown by Schmid et al. angular deviations of the foot can occur when vibrational stimuli are applied to the trunk at the level of the erector spinae muscle [62]. Trunk movements do not only involve the back; abdominal muscles are involved as well [162-164]. All of these muscular elements bear resemblance to the acupuncture meridian called girdling vessel or Dai mai vessel. Classical TCM literature attributes a holding function to this meridian. Our own imaging investigations (submitted to BMC March 2006) have led us to identify the obliquus internus muscle as the structure underlying the Dai mai point (Gb26) and the tendon of the peroneus muscle at the Shen mai point (Bl62). Punctual EMG analysis of these points during examination of movement should be feasible in specialized biomechanical laboratories.

### 6) Scintigraphic imaging data in relation to the model

**Figure 15:**
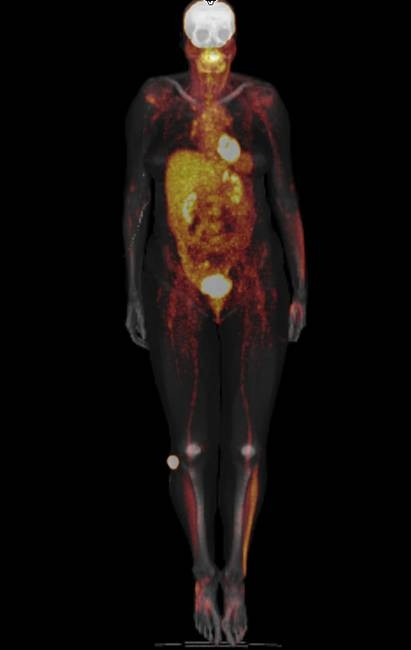
Metabolic imaging using whole body ^18^F-Fluorodeoxyglucose PET and CT. Note the metabolic activity on the lateral side of the left lower limb as well as on the right ankle. The area around the ankle corresponds to the Shen mai acupuncture point which has been evaluated in our study. Shank muscles are commonly more active in locomotion. The image was provided courteously by Dr David Townsend, University of Tennessee [165].

We would like to highlight the diagnostic possibilities of Nuclear Medicine in the context of the model. Figure 15 shows the diagnostic capabilities of combined PET-CT scanners (in this example SIEMENS, Erlangen, Germany). The fused image, i.e. superposed PET and CT whole body images, shows metabolic activation of the left lateral shank while at the same time the right ankle also shows increased metabolic activity. We interpret this image in 2 ways. In the first place it shows the anatomical localization of the changes we describe. In the second place it demonstrates the research capabilities of whole body PET-CT imaging in musculoskeletal studies with emphasis put on "whole body analysis". The image was kindly provided by Dr. David Townsend, University of Tennessee [165].

**Figure 16:**
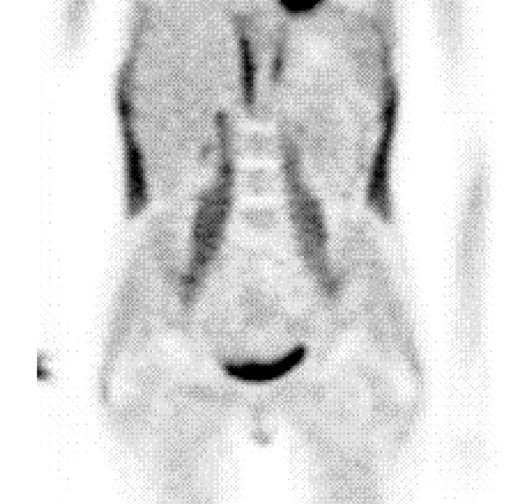
Metabolic imaging using ^18^F-Fluorodeoxyglucose (18F-FDG) showing metabolic activity in the lateral abdomen (dark areas). These areas are located around the Dai mai point which has been evaluated in our study. The accompanying CT images shows a clear deviation of the body axis at the level of the trunk. The images are reproduced with kind permission of the Society of Nuclear Medicine [166].

A second interesting image is one that shows increased metabolic activity in the lateral area of the abdomen [166]. These areas correspond to the Dai mai acupuncture point (see discussion in the preceding section) which is located below the free end of the 11 th rib. In addition to this, the morphological CT images also show the medio lateral displacement of the body axis (Figure 16). For clinical purposes, precise clinical evaluation of trunk deviation can be carried out by rasterstereography [167].

Besides the examples given here, we would like to stress the applicability of whole body ^18^F-Fluorodeoxyglucose imaging as an investigational tool to characterize whole body muscle activity in vivo [168-176] or even tendon activity [177]. In a whole body approach both agonistic and antagonistic muscle groups can be evaluated and a metabolic quantification can be done.

### 7. The WOMED concept of lateral tension, inflammation and IL-6

The WOMED concept of medical care can be summarized as an integral care of the individual. Within this concept elements of TCM and Western medicine are intermingled. Some of the evaluation parameters used in each patient are posture, locomotion, structural cohesion, previous infectious diseases, nutrition habits, nutritional status, internal medicine evaluation, and gynecological evaluation when needed. In addition to this both endocrinological and immunological aspects are included. Examples of this medical approach have been published [67,94,131].

**Figure 17:**
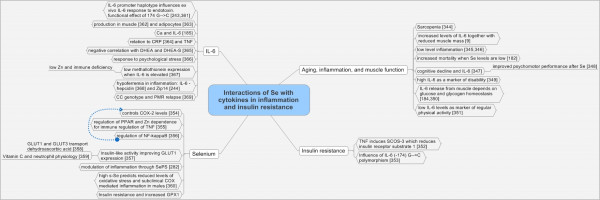
Graphical summary of scientific data related to inflammation as it can be applied in TAO and in metabolic disorders including insulin resistance. Interactions with IL-6 and Se are depicted.

Our interpretation of lateral tension involves eccentric muscle action. Due to the contained structure of the orbit with limited expansion, it does not appear plausible that the initial lesion occurs here. On the other hand, due to the frequent involvement of the ankle in daily lesions [178-180] it seems plausible that the initial pathogenic stimulus arises from this area. In addition to lesions of the lower limb, postural problems are not seldom found even in young children [181]. Therefore pre-existing postural changes together with low grade eccentric action could have a negative effect on the musculoskeletal system leading to a low grade inflammation which can also lead to consumption of vital resources such as Se and Zn. Lack of antioxidants will give way to an elevation of IL-6 [182]. The role of IL-6 in muscle metabolism has been matter of intense investigation over the past years. IL-6 appears to have several effects on the muscle and appears to play a role in the control of normal muscle function [183]. Activation of muscular IL-6 release depends also on glycogen levels [184], as well as on the interplay of Ca^2+ ^[185]. Repetitive low force exercise can also lead to increased interstitial concentrations of IL-6 [186]. The area surrounding the Achilles tendon can be taken as an example for changes seen in eccentric exercise and can be characterized by means of microdialysis studies [186-188]. This type of investigations can provide an anatomically close view of the changes that can occur. Inflammatory reactions can indeed be found by this method even when there are no apparent changes in blood [186-188]. A graphical summary of mechanisms of inflammation is given in Figure 17 pointing out the relevance of Se. Further vinculations to insulin resistance, as a part of the so-called metabolic syndrome, are also included.

The lateral tension model bears resemblance to the biomechanical five segment model of Levin et al. [189] where the feet, the legs and the abdomen are viewed as a unit. Our study has shown that the use of acupressure on the lateral abdomen normalized the TL produced by the stretch stimulus thus suggesting an important role of it for posture. Biomechanical observations have shown that a primary activation of these muscles occurs during gait [190] and that these muscles influence the mechanics and the tension of the back [191,192]. Altered foot positioning, as we have found here, can also be produced by muscle stimulation from the back [62]. This underlines the functional relationships of the ankle and trunk in locomotion [189]. The Dai mai point, located at the lateral abdomen, can be considered to be a trigger point [193] that represents the existing biomechanical tension arising from eccentric exercise. Tension will lead to taut band formation and this can be found by manual examination.

## Conclusion

The WOMED concept of lateral tension which is centered around the abdominal muscles might be applicable to other conditions of musculoskeletal diseases. It is important to point out that our proposed use of acupuncture to treat fascial changes in the WOMED model of lateral tension coincides with the expectations from Shah et al. [187]. They propose the used of microdialysis needles as surrogates for acupuncture needles to treat myofascial trigger points. By our means of manual clinical investigation, relevant myofascial trigger points can be found easily. For analytical scientific research procedures such as dynamometry, body surface scanning, and in-vivo microdialysis will provide figures and numbers related to our model. It can be expected that 2D separation of serum proteins will allow a closer insight into the changes in the proteome in selected diseases [194]. Our clinical experience in patients with TAO, multiple sclerosis, fibromyalgia and similar conditions suggests that successful treatment can rely on acupuncture techniques that aim at correcting the integration of neurophysiological inputs.

## Simple but relevant quotes

**Figure 18:**
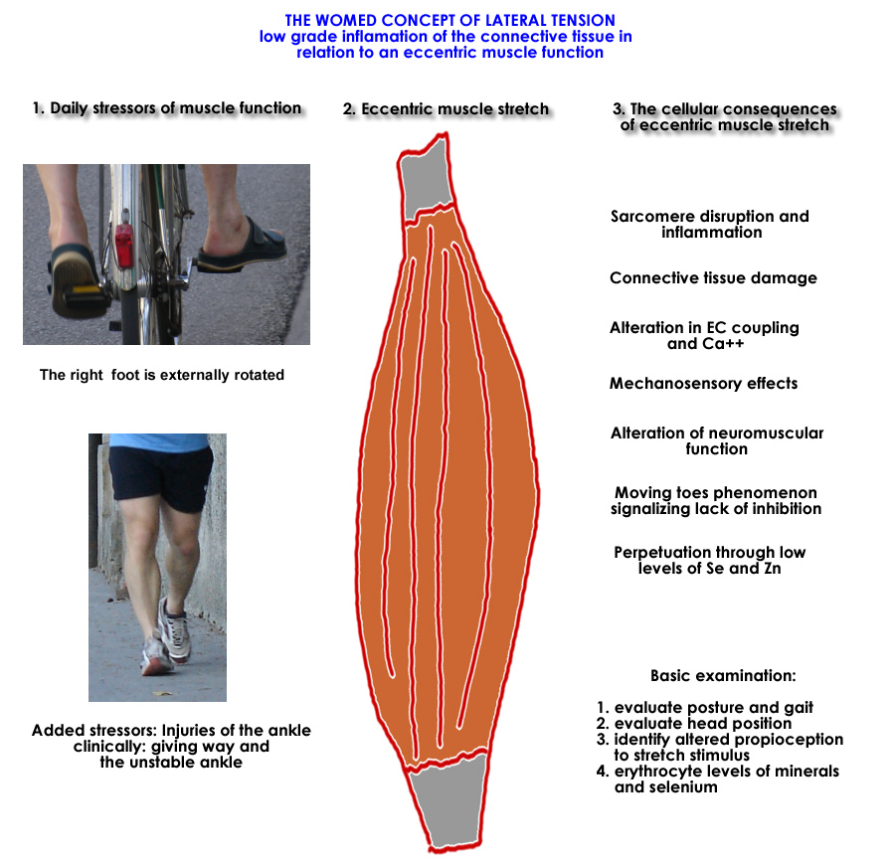
Abbridged representation of the WOMED concept of lateral tension in relation to inflammation and daily low level stressors. Further details can be extracted from figures 13, 14 and 17.

We are involved with musculoskeletal function every day and take it for granted. For this reason every day use might not appear to be relevant to disease in the first place. Terry F. Davies, Editor in Chief of Thyroid, has made reference to this situation while commenting on the pathogenesis of TAO: "It maybe very well be true that there is nothing new under the sun – it is just that we have either forgotten it or never understood it" [195]. While this applies to daily motor accomplishments, unusual musculoskeletal exercise with repetitive eccentric muscle action can produce an acute situation similar to the one we describe. The impressions of Lance Armstrong after the N.Y. marathon in November 2006 illustrates this: "Even after experiencing one of the hardest days of the Tour nothing has ever left me feeling this bad," he said at a post-race news conference. " [My shins] started to hurt in the second half, but the bigger problem the last 7 or 8 miles was the tightness in my calves and thighs. My calves really knotted up. I can barely walk right now." Figure 18 presents a graphical summary of the model together with potential every day stressors such as bicycle riding and running.

## Competing interests

The author(s) declare that they have no competing interests.

## Authors' contributions

RM and HM contributed equally to the development of the concepts of this work. RM did the art work and the Mind Maps. All authors read and approved the final manuscript.

## Note

The WOMED concept of lateral tension Changes in muscle and connective tissue due to eccentric muscle exercise [209-212]

**1 Sarcomere damage **[108,213-215]

*1.1 Potential bias in biopsy studies *[216,217]

*1.2 Weaker sarcomeres absorb more stretch *[214]

1.2.1 sarcomere damage in myofibrils [107,109]

1.2.2 Desmin, titin, junctin [218]

force reduction [219,220]

*stiffness *[221]

increased levels of CK and LDH. Increased hydroyproline and hydroxylysine excretion [222]

muscle fragments and myosin [108,223]

Desmin, titin, junctin [109]

Troponin I [224]

1.2.3 progressive weakness and low muscle tone [220,225]

1.2.4 over stretched sarcomeres [226,227]

disruption of EC [228,229]

increased number of sarcomeres [230]

failure to reconnect and shortening [231]

swelling [105,223,225,232]

stimulation of ankyrin [233]

*connection of ankyrin to obscurin and titin *[234,235]

*ECM disruption*[236]

Siehe auch: Connective tissue[42]

1.2.5 active contractile fibers turn into passive elastic tissue

altered nociceptor sensitation and neurological accomodation

passive tension [237] and mechanical laxity [180]

involvement of tendons [112,238,239]

decreased ankle ROM [76]

fascial compartments [240]

*1.3 Inflammation mediators *[104,241]

1.3.1 IL-6, TNF [15,117,242]

1.3.2 IL-6 polymorphism and Increased inflammatory response by -174 G>C [243]

1.3.3 IL-6 regulates the Zn transporter Zip-14 [244]

pro-inflammatory cytokines

1.3.4 Influence of aging [245]

1.3.5 granulocytes and neutrophils [106,246]

1.3.6 c-Fos and lipocortin II [215]

1.3.7 induction of histidine decarboxylase [118,119]

1.3.8 increased levels of bradykinin [116]

1.3.9 interactions in nociception [247]

1.3.10 ubiquitin proteosome proteolysis [246]

1.3.11 role of gender [248]

1.3.12 Antioxidants diminish cytokine response [249]

1.3.13 activation of the c-Jun NH(2)-terminal kinase (JNK) [250]

*1.4 Elements of Ca regulation *[251,252]*and EC coupling *[253]

1.4.1 reduced Ca levels [253,254]

1.4.2 ryanodine receptor, calmodulin [251,255]

1.4.3 calsequestrin [42,133,253]

1.4.4 parvalbumin (in motoneuron) [137]

excitoxicity [256]

1.4.5 Ca, SERCA [254]

SERCA 1 in fast twitch fibers [251]

SERCA 2 in slow twitch fibers [251]

SERCA in EOM [257]

Relation to thyroid hormones [258,259]

Relation to extraocular muscles [257]

1.4.6 Triadin [253]

1.4.7 Junctin in myocyte [218]

1.5 Muscle repair processes

1.5.1 mTOR [260,261]

1.5.2 Satellite cells [262-264]

1.5.3 Zn, MMP and remodelling [265,266]

1.5.4 Telomerase in fatigue associated myopathy [267]

**2 Connective tissue **[236]

*2.1 mechanosensory effects *[268,269]

*2.2 mechanosensation of the skin *[270]

2.3 Structural changes

2.3.1 SOD changes ? [271]

2.3.2 release of glycosaminoglycans ? [27]

2.3.3 GAGs in ER [26]

2.3.4 Scarred fascia and GAGs [25]

2.4 interactions with collagen

2.4.1 collagen degrading enzymes: TIMP-1, TIMP2- MMP2, MMP-9 [110,265,272,273]

2.4.2 procollagen processing [111]

*2.5 Potential interaction with fasciae *[240]

*2.6 increased levels of bradykinin and adenosine *[115,116]

**3 Neuromuscular dysfunction **[120,122,124,125]

*3.1 Reduced reflex sensitivity *[121]

*3.2 Alpha motoneuron pool recruitment *[122]

*3.3 Alteration in sense of effort and motor command *[123]

*3.4 C-FOS expression in anterior pretectal nucleus in relation to pain *[274]

*3.5 C-FOS in the dorsal horn due to compression *[275]

*3.6 sensory control and inflammatory mediators *[247]

*3.7 Neuromuscular activity modulates myonuclear domain size *[263]

*3.8 Nervous system strategies *[276]

3.8.1 Eccentric exercise and brain activation

3.8.2 Brain activation and ECC

3.8.3 Brain control of coordinated movements

4 Nutritional and endocrine factors

4.1 Mg and Ca

4.1.1 Decrease of the ion dependent binding of spin labeled ATP [277]

*4.2 Selenium *[55]

4.2.1 Decreased levels in thyroid disease (Moncayo 2006)

4.2.2 Se in the pituitary [278]

4.2.3 Se and muscle [279]

4.2.4 Se and heart [280]

4.2.5 Se, mTOR and muscle hypertrophy [281]

4.3 Selenoproteins

4.3.1 Selenoprotein S influences inflammatory response [282]

4.3.2 Selenoprotein K – a cardiac antioxidant [280]

4.3.3 Selenoprotein N – muscular dystrophies [283]

4.3.4 Selenoprotein N1 – insulin resistance [284]

4.3.5 Selenoprotein W and muscle growth [285]

4.4 Zinc

4.4.1 Calsequestrin [133]

4.4.2 Bradykinin [286]

4.4.3 Zn and Cu and Ca binding proteins [287]

4.4.4 Influence on TRH processing [288]

4.4.5 Zn and MMP in muscle repair [265]

4.4.6 Zn and mTOR [289]

4.4.7 Synaptic activity [290]

4.4.8 Inhibitory activity in the spinal cord [146]

4.4.9 Sciatic nerve lesions and Zn transport [291]

4.5 Se and Zn

4.5.1 Zn-Se nanocrystals in the pituitary [292]

*4.6 Vitamin E *[293]

*4.7 Thyroid hormones *[294,295]

4.7.1 Regulation of muscle gene expression [294]

4.7.2 SERCA [259,296]

4.7.3 SERCA and thermogenesis [258]

4.7.4 Satellite cells [297]

4.7.5 Change in Myosin and SERCA isoforms [295]

4.7.6 Force reduction [298]

4.7.7 Changes in Ca uptake [299]

*4.8 Insulin resistance *[300,301]

*4.9 Altered glycogen accumulation *[302]

5 Muscle action of extraocular muscles

5.1 Consideration of muscle volume

5.1.1 EOM [100,101]

5.1.2 Volume of limb and abdominal muscles [102,103]

*5.2 Inapparent TAO *[303,304]

*5.3 Thyroid hormone receptors in EOM *[305,306]

*5.4 Satellite cells *[307,308]

5.4.1 Activated satellite cells in overacting EOM [309]

*5.5 Alterations in gaze *[310]

*5.6 SERCA in extraocular muscles*[257]

*5.7 Muscle overaction and Ca uptake *[127]

*5.8 Neck vibration in strabismus *[311]*associated with changes in congruent muscle propioceptive information*

*5.9 Head and eye movement in MRI *[312]

*5.10 Influence of waist disturbance on gait *[313]

*5.11 Ocular motility *[314]

*5.12 Layer specific EOM structure *[128,315-317]

6 Role of brain integration in movement coordination

*6.1 Interlimb coordination *[318]

*6.2 Lateralization of lower limb movements *[151]

*6.3 Coordination of ankle movements *[150]

*6.4 Active and passive ankle movements *[319]

***Regulation of Ca Release *** [320]

**1. Ca cycling **[254]

2. SERCA

2.1. SERCA 1 fast fibers

2.1.1. Sarcolipin (SLN) [321,322]

2.1.2. Brody disease [320]

2.2. SERCA 2 slow fibers

2.2.1. Phospholamban (PLB) [321,322]

3. Calsequestrin (high capacity – low affinity)

3.1. CSQ1 fast

3.2. CSQ2 slow

3.2.1. CSQ2 deletion > catecholaminergic tachycardia [323]

3.3. β Dystroglygan and CSQ assembly

**4. Calreticulin **[263]

*4.1. cardiac myofibrillogenesis*[324]

**5. Na Ca exchanger **[325]

**6. IP3 **[43,326]

*6.1. Involvement in redox echange regulation *[326]

**7. Dihydropyridine receptor DHPR **[251]

7.1. Voltage sensor

*7.2. Linked to CSQ via junctional protein 45 *[327]

7.3. Subtype 1

7.3.1. Malignant Hyperthermia

8. Ryanodine 3 gene products and splicing variants

8.1. RYR1 skeletal

8.1.1. minicore ophthalmoplegia

8.1.2. multi minicore disease

8.1.3. central core disease

8.1.4. malignant hyperthermia

8.2. RyR2 cardiac

8.2.1. arrhythmias

8.3. RyR3 cephalic muscles

*8.4. Regulation by thiols *[328]

*8.5. Importance of glutathione *[329]

*8.6. Importance of Se *[330]

*8.7. Junctin in the structure *[331]

9. Ca buffers

*9.1. Parvalbumin *[41]

9.2. Troponin C

*9.3. Calmodulin *[332]

9.4. Histidin-rich calcium binding protein

9.5. Junctate

9.6. Sarcolumenin

9.7. Mitochondria

*9.8. S100 *[333]

**10. Role of thyroid hormones **[334]

10.1. Hyperthyroidisim

10.1.1. Upregulates Na Ca exchanger

10.1.2. IP3

10.1.3. RyR

10.1.4. Myosin heavy chains

10.1.5. Gender specific action via βERB

10.1.6. Low levels of Zn [79]

**11. Role of Selenium and selenoproteins **[279]

11.1. Regulates functional thiols of RyR

11.1.1. oxidation > open

11.1.2. reduction > closed

*11.2. Selenoprotein N *[335]

11.2.1. Related to rigid spine [283]

11.2.2. Minicore ophthalmoplegia [336]

11.3. Selenoprotein K

11.3.1. Cardiac antioxidant [280]

*11.4. Selenoprotein W *[337,338]

11.4.1. Oxidative stress [285]

12. Role of Zn

*12.1. Modulation of activation and inactivation of Ca on RyR *[339]

*12.2. Associated with T-tubules *[340]

*12.3. CSQ structure *[133,135]

12.4. Ca Zn binding to calmodulin

*12.5. Ca Zn and MMP *[341]

*12.6. HRP regulation *[135]

*12.7. Higher levels in red muscle *[342,343]

Interactions of Se with cytokines in inflammation and insulin resistance

1. Aging, inflammation, and muscle function

*1.1. Sarcopenia *[344]

*1.2. increased levels of IL-6 together with reduced muscle mass *[9]

*1.3. low level inflammation *[345,346]

*1.4. increased mortality when Se levels are low *[182]

*1.5. cognitive decline and IL-6 *[347]

1.5.1. improved psychomotor performance after Se [348]

*1.6. high IL-6 as a marker of disability *[349]

*1.7. IL-6 release from muscle depends on glucose and glycogen homeostasis *[184,350]

*1.8. low IL-6 levels as marker of regular physical activity *[351]

2. Insulin resistance

*2.1. TNF induces SCOS-3 which reduces insulin receptor substrate 1 *[352]

*2.2. Influence of IL-6 (-174) G–>C polymorphism *[353]

3. Selenium

*3.1. controls COX-2 levels *[354]

*3.2. regulation of PPAR and Zn dependence for immune regulation of TNF *[355]

*3.3. regulation of NF-kappaB *[356]

Siehe auch: controls COX-2 levels

*3.4. Insulin-like activity improving GLUT1 expression *[357]

3.4.1. GLUT1 and GLUT3 transport dehydroascorbic acid [358]

3.4.2. Vitamin C and neutrophil physiology [359]

*3.5. modulation of inflammation through SePS *[282]

*3.6. high s-Se predicts reduced levels of oxidative stress and subclinical COX mediated inflammation in males *[360]

3.7. Insulin resistance and increased GPX1

4. IL-6

*4.1. IL-6 promoter haplotype influences ex vivo IL-6 response to endotoxin. functional effect of 174 G–>C *[243,361]

*4.2. production in muscle *[362] and adipocytes [363]

*4.3. Ca and IL-6 *[185]

*4.4. relation to CRP *[364]*and TNF*

*4.5. negative correlation with DHEA and DHEA-S *[365]

*4.6. response to psychological stress *[366]

*4.7. low methallothionein expression when IL-6 is elevated *[367]

4.7.1. low Zn and immune deficiency

*4.8. hypoferremia in inflammation: IL-6 – hepcidin *[368]*and Zip14 *[244]

*4.9. CC genotype and PMR relapse *[369]

## Pre-publication history

The pre-publication history for this paper can be accessed here:



## Figures and Tables

**Table 1:** Outline of the WOMED clinical examination protocol

**Position**	**Parameter**	**Characteristics**
**Sitting or standing**	Eyes in orbit	Symmetry, angular displacement
	Orbit on head	Symmetry, angular displacement
	Head on neck	Rotation, tilting
	Neck on trunk	Rotation, tilting, tender points
**Standing**	Hip	Medio lateral height symmetry

**Unloaded supine position**	Leg axis	Symmetrical position, axial rotation
	Feet on shank	Extension, inversion

**Functional propioceptive**	Foot inversion	Changes in the test muscle
**test**	Foot rotation	Moving toes phenomenon

**Table 2:** Comparison of erythrocyte mineral and metal levels

**Study Groups**		**Ca**	**Mg**	Zn*	**Cu**	**Mg Ca ratio**	Cu Zn ratio**
**TAO patients**	n	13	13	13	13	13	13
	Mean	60,78	33,82	6,75	1,42	0,56	0,22
	S.D.	3,11	2,31	1,22	0,48	0,04	0,08
	min.	56,89	30,14	4,30	1,02	0,50	0,13
	max.	68,65	37,29	8,57	2,82	0,62	0,40

**Normal controls**	n	16	16	16	16	16	16
	mean	60,73	33,41	7,41	1,25	0,55	0,17
	S.D.	1,83	3,01	0,46	0,32	0,06	0,04
	min.	56,70	29,19	6,40	0,90	0,47	0,12
	max.	64,45	38,14	8,17	1,96	0,67	0,27

* p = 0.05, ** p < 0.05

**Table 3:** Correlation analysis of Zn, Ca, Mg and Cu levels

			Mg	Cu	Zn	Fe
TAO	Ca	r	0,239	0,641*	-0,060	-0,758**
		p	0,432	0,018	0,845	0,004
		n	13	13	13	12

	Mg	r		-0,042	0,121	0,125
		p		0,892	0,693	0,699
		n		13	13	12

	Cu	r			0,116	-0,663*
		p			0,706	0,019
		n			13	12

	Zn	r				-0,165
		p				0,609
		n				12

Controls	Ca	r	-0,632**	0,357	-0,085	-0,485
		p	0,009	0,175	0,756	0,057
		n	16	16	16	16

	Mg	r		-0,349	-0,097	0,625**
		p		0,186	0,721	0,010
		n		16	16	16

	Cu	r			0,094	-0,347
		p			0,730	0,188
		n			16	16

	Zn	r				-0,277
		p				0,300
		n				16

* The correlation is significant at a level of 0,05 (2-sided).

** The correlation is significant at a level of 0,01 (2-sided).

**Table 4:** Postural changes observed in TAO patients and implications of these changes

**Basic structure**	**Related organ**	**Description of the alteration**	**Axes**	**Potential effects**
Head	Eyes	Elevation, tilted	X, Y, Z	postural alignment [196,197]
	Neck	Rotated, tilted, extended or flexed	X, Y, Z	dorsal muscles [198] ocular torticolis [96,97]
	Nose	Tilted	Y, Z	Retro nasal space?
	Mouth	Tilted	Y	Jaw [199]
Face		Asymmetry		Head holding [200]
Neck	Trunk	Rotated	Y	Lymphatics, posture [200,201]
Trunk and abdomen		Rotated Increase of the intra-abdominal pressure	Y	Foot angle [62] Pelvic girdle pain [202]
Leg	Pelvis Gluteus m.	Rotated	Y	Hip sway Transversus abdominis [190] Altered femoral control [203]
Ankle	Shank Hip	Reduced range of motion	X, Y, Z	Instability [204], Sway Sensory changes and hypermobility [205,206] Altered hip load – unload [126] Hip weakness [207]
Feet	shank	Lateral displacement	Y, Z	Tightness of lateral calf muscles [156]
Feet	Sole	Asymmetry	Y	Posture information [155] Influence on retroocular muscles [55]
Foot to head	Whole body			Posture asymmetry [208]
